# Tryp: a dataset of microscopy images of unstained thick blood smears for trypanosome detection

**DOI:** 10.1038/s41597-023-02608-y

**Published:** 2023-10-18

**Authors:** Esla Timothy Anzaku, Mohammed Aliy Mohammed, Utku Ozbulak, Jongbum Won, Hyesoo Hong, Janarthanan Krishnamoorthy, Sofie Van Hoecke, Stefan Magez, Arnout Van Messem, Wesley De Neve

**Affiliations:** 1 Center for Biosystems and Biotech Data Science, Ghent University Global Campus, Incheon, 21985 South Korea; 2 IDLab, Ghent University, Technologiepark-Zwijnaarde 126, B-9052 Ghent, Belgium; 3 IDLab, Ghent University - imec, Technologiepark-Zwijnaarde 126, B-9052 Ghent, Belgium; 4 School of Biomedical Engineering, Jimma Institute of Technology, Jimma University, Jimma, Ethiopia; 5 Biomedical Research Center, Ghent University Global Campus, Incheon, 21985 South Korea; 6 Laboratory of Cellular and Molecular Immunology, Vrije Universiteit Brussel, Brussels, Belgium; 7 Department of Biochemistry and Microbiology, Ghent University, Ghent, Belgium; 8 University of Liège, 4000 Liège, Belgium

**Keywords:** Machine learning, Infectious-disease diagnostics

## Abstract

Trypanosomiasis, a neglected tropical disease (NTD), challenges communities in sub-Saharan Africa and Latin America. The World Health Organization underscores the need for practical, field-adaptable diagnostics and rapid screening tools to address the negative impact of NTDs. While artificial intelligence has shown promising results in disease screening, the lack of curated datasets impedes progress. In response to this challenge, we developed the Tryp dataset, comprising microscopy images of unstained thick blood smears containing the *Trypanosoma brucei brucei* parasite. The Tryp dataset provides bounding box annotations for tightly enclosed regions containing the parasite for 3,085 positive images, and 93 images collected from negative blood samples. The Tryp dataset represents the largest of its kind. Furthermore, we provide a benchmark on three leading deep learning-based object detection techniques that demonstrate the feasibility of AI for this task. Overall, the availability of the Tryp dataset is expected to facilitate research advancements in diagnostic screening for this disease, which may lead to improved healthcare outcomes for the communities impacted.

## Background & Summary

Trypanosomiasis is a debilitating disease caused by pathogenic species of the trypanosome parasite. The World Health Organization (WHO) has categorized two forms of this condition, namely Chagas disease and human African trypanosomiasis (HAT), as neglected tropical diseases (NTDs)^1,2^. Chagas disease, also known as American trypanosomiasis, is caused by the parasite *Trypanosoma cruzi* and is primarily transmitted by infected triatomine bugs. This disease is mainly found in Latin America, affecting approximately six million individuals worldwide^3^. HAT, commonly referred to as sleeping sickness, is caused by two species of the *Trypanosoma brucei* parasite, namely *T. b. gambiense* and *T. b. rhodesiense*. Tsetse flies in sub-Saharan African nations are the primary vector for HAT transmission. If left untreated, HAT is usually chronic and fatal, with infected individuals frequently succumbing within six months^4^.

NTDs exert devastating human, social, and economic burdens on over one billion people worldwide, causing approximately 200,000 fatalities each year^5^. This impact is especially concerning as it disproportionately affects the most impoverished, vulnerable, and marginalized populations, impeding the achievement of the third United Nations Sustainable Development Goal (SDG) of ensuring good health and well-being. To end the neglect of attaining the SDGs, the WHO 2021–2030 roadmap for NTDs has identified the development of effective field-adaptable diagnostics and rapid screening tools as a prerequisite for meeting their trypanosomiasis targets by 2030^5^.

Despite its prevalence in the screening and diagnosis of trypanosomiasis, manual microscopy presents notable limitations, including its labor-intensive nature, low sensitivity, and the requirement for skilled personnel^5–8^. Firstly, the labor-intensive nature of manual microscopy necessitates a significant commitment of time and resources, potentially causing delays in diagnosis and treatment in settings with high disease prevalence. Secondly, the inherent subjectivity of the approach can lead to inconsistencies in result interpretation, thereby compromising the sensitivity and overall diagnostic accuracy of the technique. Lastly, the necessity for skilled personnel, particularly problematic in resource-constrained environments where the disease is endemic, can significantly impede effective disease screening and diagnostic practices due to limited access to trained professionals^5^. We posit that integrating Artificial Intelligence (AI) could substantially alleviate the aforementioned challenges inherent to manual microscopy in trypanosomiasis diagnosis. The potential application of AI to screen or diagnose diseases is promising and is receiving increasing research attention^9–15^. Researchers have also employed AI to detect or screen NTDs such as trachomatous trichiasis^16^, leprosy^17^, helminths and schistosoma^18^, and trypanosomiasis^19,20^. While the current body of research on using AI for automated screening of trypanosomiasis from microscopy images of fresh unstained thick blood smears is relatively sparse, the choice to utilize unstained fresh blood samples was a deliberate one, informed by the urgent needs of prominent research laboratories in the field of trypanosomiasis research. This approach, which emphasizes efficiency and innovation, aims to obviate the need for staining techniques, potentially transforming the method by which parasites are identified in practice.

To address the limitations of manual microscopy, we have created a curated dataset for detecting trypanosome parasites in microscopy images of unstained thick blood smears. Our dataset enables the training of deep learning models to detect the trypanosome parasite in these images. We further provide a benchmark on three leading deep learning-based object detection techniques that demonstrate the feasibility of AI for this task. This way, we want to stimulate AI research on trypanosome parasite detection to help facilitate the achievement of the WHO targets.

## Methods

**Fig. 1:**
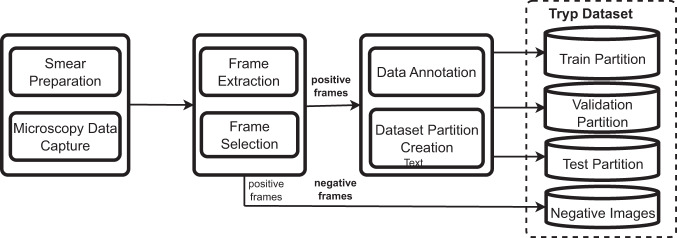
Flow diagram of the Tryp dataset creation process.

The *Tryp* dataset has been curated to facilitate research on developing and assessing object detection models specifically tailored for trypanosomiasis screening. As visually summarized in Fig. 1, this section details the comprehensive procedures and methodologies employed in generating and characterizing this dataset.

### Thick blood smear preparation

Eight-week-old female C57BL/6 mice were purchased from Koatech (Gyeonggi-do, Republic of Korea) and infected by intraperitoneal injection using 5 × 10^3^ *T. b. brucei AnTat1.1E*. All experiments were approved by the Institutional Animal Care and User Committee (IACUC) of the Ghent University Global Campus under the approval numbers GUGC-IACUC-2021-005 and GUGC-IACUC-2021-009. Thick-smear blood samples were prepared by taking a tail snip blood drop, placing it onto a microscopy glass, and covering it with a microscopy cover slip by gently applying pressure. Samples were always collected from mice that were part of other ongoing laboratory research experiments, and no animals were sacrificed specifically for this study.

### Microscopy data acquisition

**Fig. 2:**
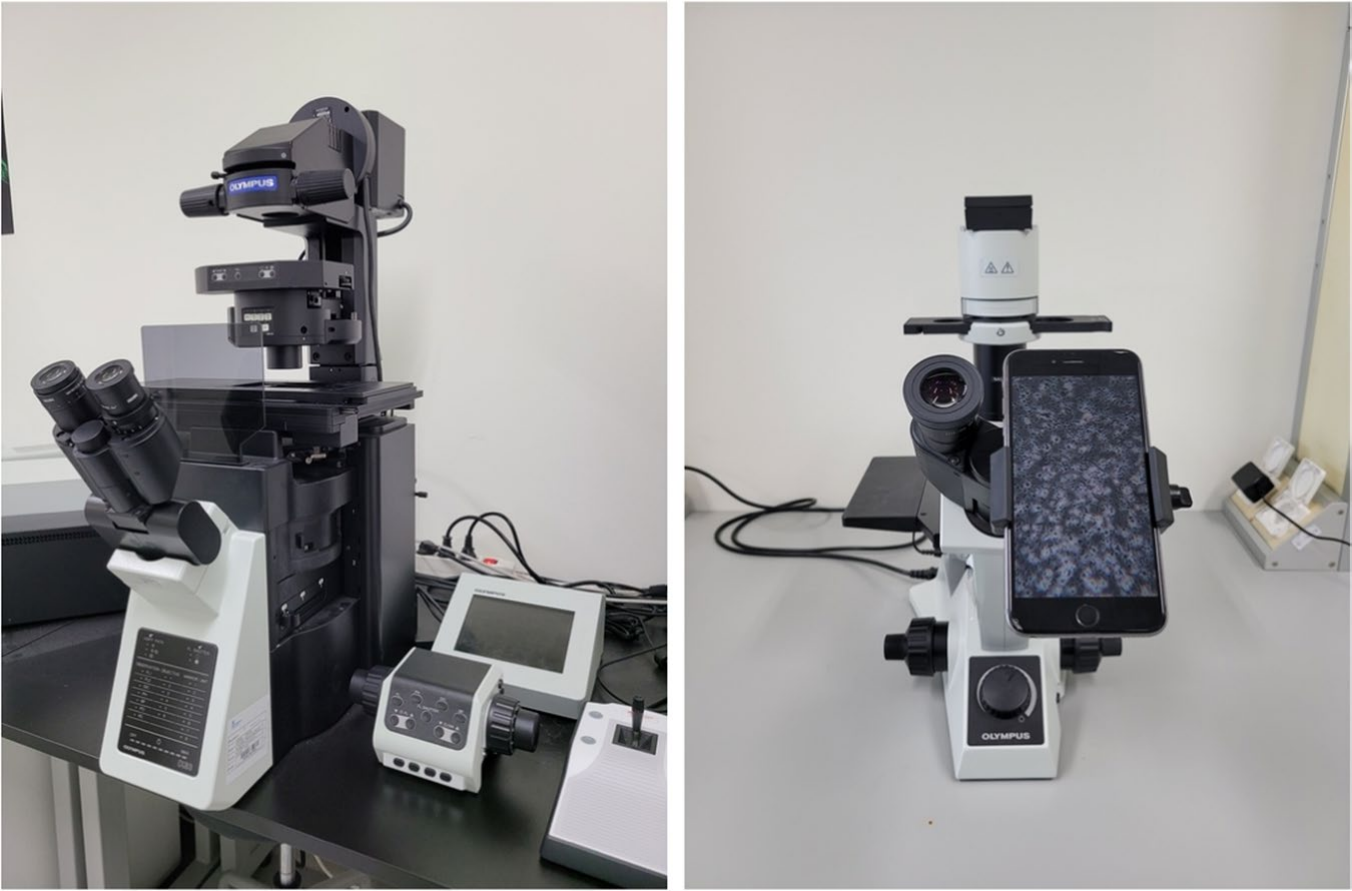
The microscopy video capture devices used to obtain the videos used in Tryp: an IX83 inverted Olympus microscope (left) and a simple manual Olympus CKX53 microscope (right).

Over multiple days, ten student researchers with diverse expertise, alongside a trypanosomiasis research expert, captured microscopy images in video sequences using two distinct Olympus microscope setups, shown in Fig. 2. Additional specifications for the two setups are provided in Table 1. While the IX83 microscope has built-in video capture capability, mobile phones were attached directly to the eyepiece of the CKX53 microscope to enable video capture. The models of the mobile phones used are iPhone 6, 6 S Plus, 12, and Samsung Galaxy Note 10. The video acquisition process resulted in 103 videos of infected blood samples and 11 videos of non-infected blood samples.

**Table 1:** Microscope Specifications and Video Capture Resolutions.

Microscope	Magnification	Objective	Numerical Aperture	Configuration	Video Capture Mechanism	Video Resolution
IX83	40x	LUCPlanFL N	0.60	DIC	Inbuilt	1,360 × 1,024
CKX53	20x	LCAchN	0.40	iPC US2 Phase-contrast	An attached Mobile Phone	1,920 × 1080, 720 × 404

**Fig. 3:**
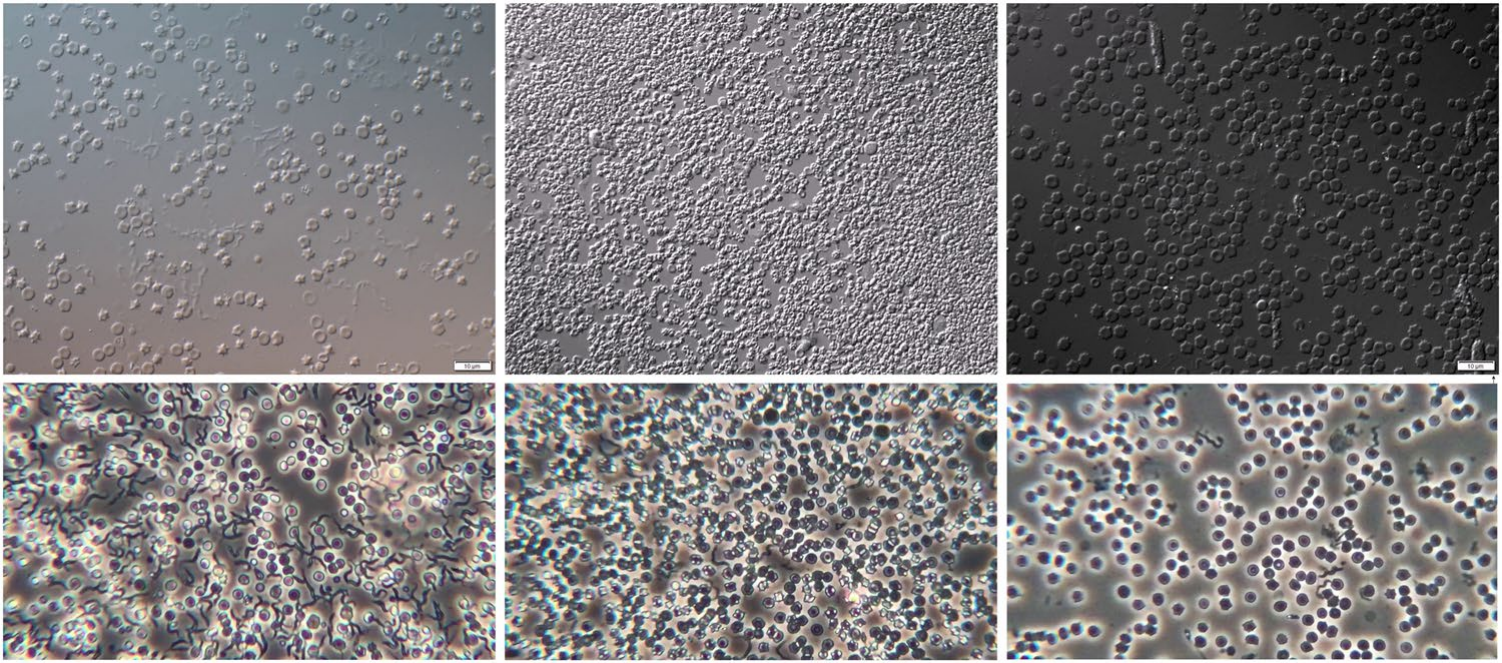
Randomly selected example images captured with the IX83 inverted Olympus microscope (top row) and Olympus CKX53 microscope (bottom row). The images of the microscopes used are shown in Fig. 2.

Data quality is crucial in developing deep neural network (DNN) models for real-world applications, especially in critical areas such as health care. To ensure that the data acquisition process closely reflects real-world scenarios, we implemented specific measures, such as using thick blood smears that allow parasites to move in and out of visibility within the same microscope field of view (FOV). Additionally, we encouraged the expert and student researchers to (1) freely use the microscope settings that help them to confirm the presence or absence of parasites within the microscope FOV without any restrictions and (2) cover multiple FOVs in a single thick blood smear whenever possible. We provide a small sample of the extracted frames in Fig. 3 to illustrate the diversity in the capturing process.

### Frame extraction and selection

#### Frame extraction

The models evaluated in this study take images as input, requiring the conversion of captured videos in formats with extensions such as *.mov*, *.avi*, and *.mpeg4* into a series of JPEG image frames, resulting in 40,931 images. However, the annotation of such a large number of images is cost-prohibitive, and the video capture process introduces limitations, including temporal redundancy and motion blur, which can diminish the effectiveness of certain frames for training DNN models. Temporal redundancy may arise in microscopy video capture of trypanosome parasites due to the fixed position of the microscope eyepiece and the smear slide, resulting in consecutive frames with minimal changes, despite the high motility of the parasites.

#### Frame selection

**Fig. 4:**
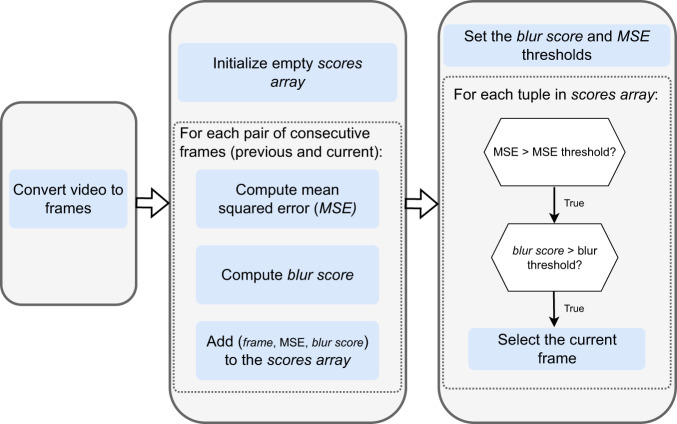
A summary of the process for selecting the extracted frames to be included in the Tryp dataset.

To overcome the challenges outlined in the previous paragraph and reduce the annotation effort required, a procedure for the selection of frames to be included in the Tryp dataset was implemented. This procedure begins with the conversion of video files into a series of JPEG image frames. Subsequently, the mean squared error (MSE) between consecutive frames is calculated, along with the variance of the Laplacian to derive a blur score. These steps apply two simple computer vision techniques–motion blur check and frame differencing. Setting the stage for the critical process of frame selection, thresholds for both the MSE and blur scores are established, and only frames that exceed these thresholds are retained. The resulting set of 3,085 representative frames is chosen to be included in the dataset. This entire process, executed using the Python programming language and the OpenCV library from https://opencv.org, reflects a methodical approach to frame selection that is underpinned by quantitative metrics. Figure 4 presents a visual overview of the processes described above.

### Data annotation

**Fig. 5:**
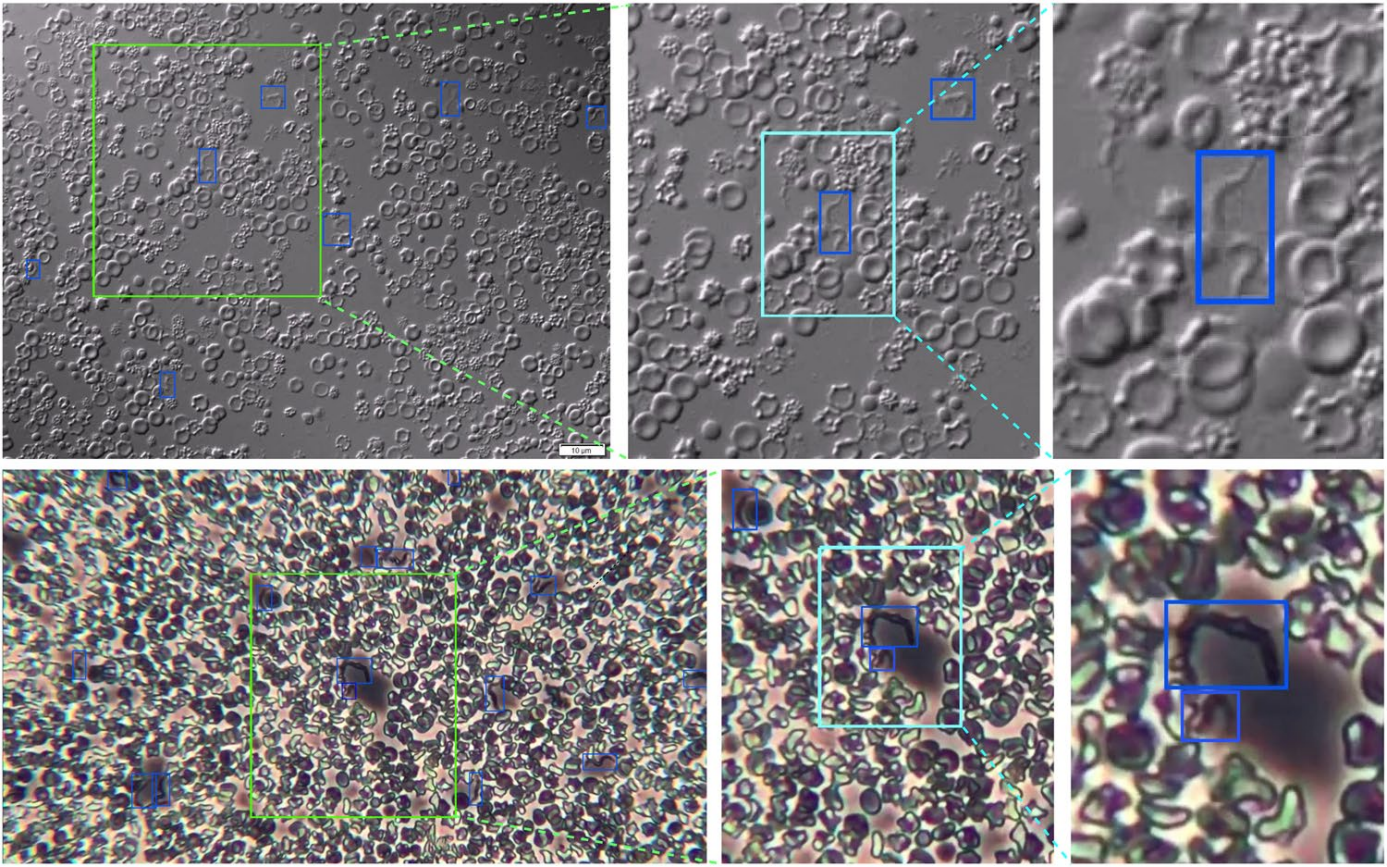
Two example microscopy images and zoomed regions to show the trypanosome parasites, their size compared to blood cells, and how the bounding boxes are tightly drawn around them. In all images, blue boxes indicate bounding boxes created with the procedure discussed in the sub-section “Data Annotation”. Images in the top row are obtained with the IX83 inverted Olympus microscope, while those at the bottom are obtained with the Olympus CKX53 microscope.

**Fig. 6:**
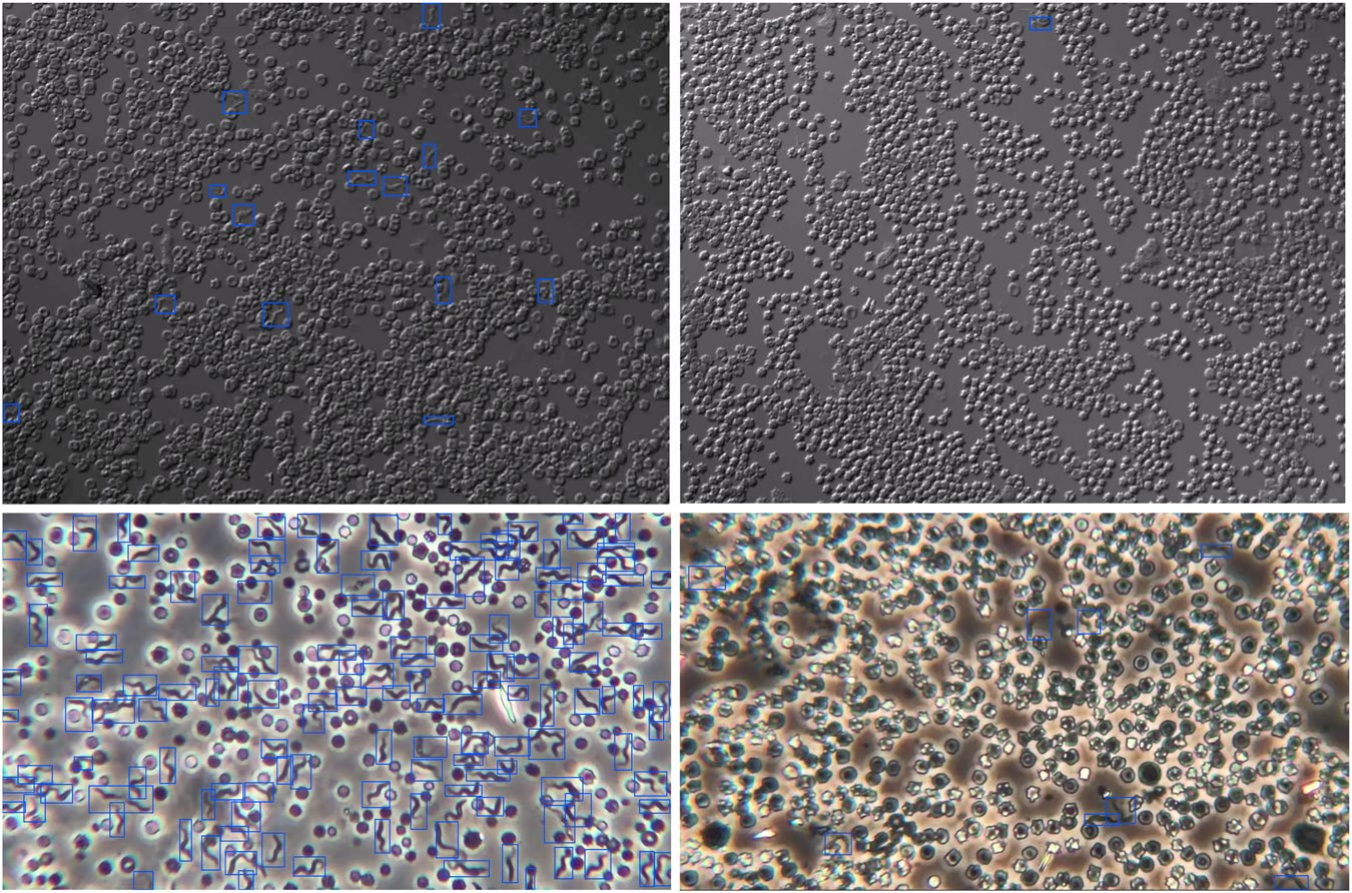
Example microscopy images obtained with the IX83 inverted Olympus (top row) and Olympus CKX53 (bottom row) microscopes. The left column images show microscopy images of blood smears containing many parasites, while those with fewer parasites are shown on the right column.

The annotation process involves defining a rectangle around observable parasites within microscopy images as illustrated in the magnified regions of the images in Fig. 5. Figure 6 provides additional examples, demonstrating the variation in trypanosome parasite concentration. We used two platforms for the annotation process: the online platform, Roboflow (https://roboflow.com), and the open-source platform Labelme (https://github.com/wkentaro/labelme. In both platforms, users draw tight bounding boxes around parasites, and the two coordinates (top left and bottom right) that fully describe the bounding boxes are automatically recorded.

Roboflow enables the export of created annotations in various formats, including Microsoft Common Objects in Context (MS COCO) and text files, catering to our specific needs. For Labelme, we utilized its unique JavaScript Object Notation (JSON) file format for the annotations of each image. Since our models required specific formats, we developed custom Python scripts to convert the Labelme annotations into the MS COCO and text formats required for our experiments. We provide illustrative images in Fig. 6 to show examples of tight bounding boxes around parasites and variations in parasite density. Some captured microscopy images contain many visible parasites, while others contain fewer parasites.

Following best practices in object annotation, ten annotators engaged in a two-stage annotation process to maintain a consistent and unified standard. In the initial stage, seven annotators created preliminary annotations. Subsequently, a separate group of three annotators, with a specific focus on maintaining the consistency and quality of annotations, rigorously verified and rectified any discrepancies, including missing or inadequately defined bounding boxes. This dual-stage process was implemented to ensure the highest accuracy and consistency in our annotation effort, recognizing the importance of good quality annotations for training robust deep learning models.

### The Tryp datset partitioning and bounding box characteristics

The dataset comprises four partitions: train, validation, test, and “negative images”. The train, validation, and test partitions contain annotated microscopy images of infected blood samples. In contrast, the “negative images” partition consists of non-annotated microscopy images from non-infected blood samples, serving as control samples for additional evaluation. In the following paragraphs, we will provide a detailed description of both the annotated and non-annotated dataset partitions.

#### Annotatated dataset partitioning

The dataset comprises 3,085 microscopy images, each depicting blood samples infected with trypanosomes. We employed two strategies to partition the images in the Tryp dataset: Stratification by Video Frames (SVF) and Stratification by Entire Videos (SEV). The SVF method divides the dataset at the frame level, facilitating a balanced data distribution across different partitions. However, this strategy carries the risk of producing overly optimistic performance outcomes. This is because the model is evaluated using distinct frames from videos partially exposed during training, which may inadvertently share unobserved characteristics. On the other hand, the SEV method allocates each video to a single partition, thereby eliminating any overlap. Implemented within a cross-validation framework, this strategy precludes data leakage, upholds inter-frame correlations, and guarantees that the model is evaluated using entirely unrelated sets of videos. This, in turn, fosters a more rigorous evaluation of the capabilities of the model. Following the SVF approach, we randomly allocated the images into three separate partitions: train (1,893 images), validation (610 images), and test (612 images), maintaining an approximate ratio of 60:20:20. Figure 1 visually illustrates the process adopted to construct the dataset using the SVF approach. The detailed distribution of the images using the SVF method is shown in Table 2. For the SEV approach, we employed a 5-fold cross-validation evaluation methodology. The attributes of the folds are detailed in Table 3. The evaluation results for both strategies are detailed in the Results section. For the rest of the paper, mentioning the Tryp dataset without explicitly mentioning SEV refers to the partitions created using the SVF partitioning strategy

#### Characteristics of annotated bounding boxes

Here, we delve into the characteristics of the generated bounding box annotations, and explore metrics that provide quantitative insights into the parasite density, spatial relationships, and the overall parasite distribution within the images. We briefly describe these metrics and present their histogram plots.

##### Bounding box count per image

The bounding box count per image represents a straightforward yet insightful metric, quantifying the number of bounding boxes annotated in each image. This count aids in assessing the complexity and diversity of the parasites within the image, providing essential information about parasite density and potential detection challenges. Differentiating between images with high and low bounding box counts offers valuable cues for tailoring detection algorithms to suit specific characteristics of parasite images.

##### Overlapping bounding box count per image

This metric quantifies the number of pairwise overlaps between bounding boxes within an image, where an overlap is defined as a spatial intersection between two bounding boxes. For example, if bounding box A overlaps with B, and B overlaps with C, but A and C do not overlap, the count would be two. Understanding the prevalence of such overlaps helps assess the complexity of spatial arrangements within the dataset, offering a nuanced perspective on potential challenges in parasite detection.

##### Region-of-Interest (RoI) ratio

In this work, we introduce the RoI ratio metric to quantify the ratio of the combined area of bounding boxes to the total area of an image. This quantity specifically considers the union of all bounding boxes within an image, thereby ensuring that overlapping regions are counted only once. The RoI ratio offers additional insight into the spatial distribution and density of parasites within images in the dataset and could aid in better understanding the characteristics of datasets.

**Fig. 7:**
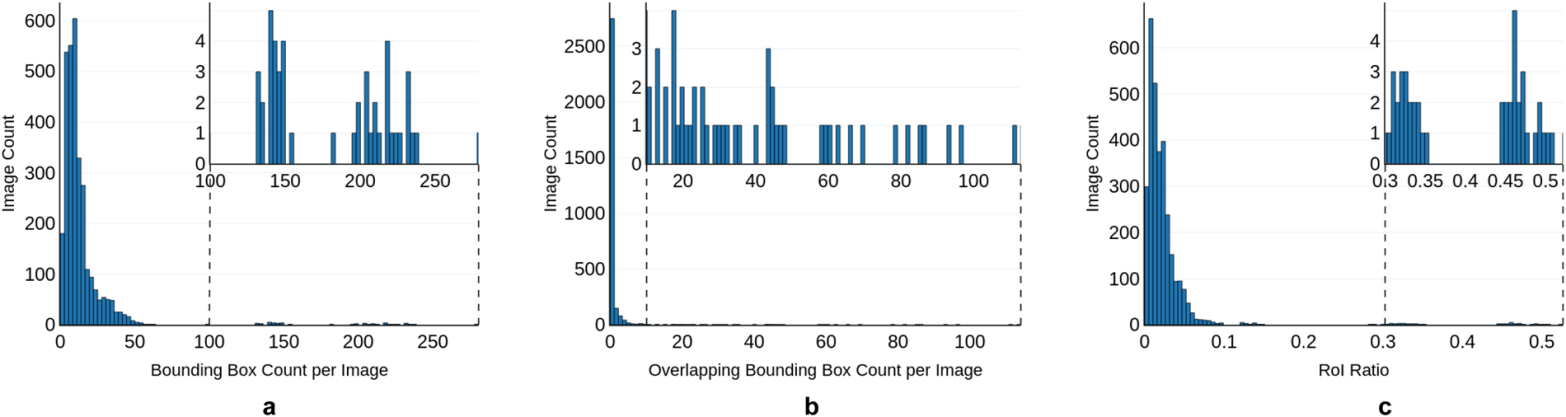
Histograms for three characteristics of the ground truth bounding box annotations for the Tryp dataset. These characteristics are: (**a**) the bounding box count per image, (**b**) the overlapping bounding box count per image, and (**c**) the RoI ratio.

The distributions of the bounding box count per image, overlapping bounding box count per image, and the RoI ratio can be found in Fig. 7, considering all the bounding boxes in the Tryp dataset. These histograms allow for a visual examination of the characteristics and trends of the annotated parasite bounding boxes within the Tryp dataset. We set the IoU to be at least 0.1 for two bounding boxes to be considered as overlapping.

#### Non-annotated dataset partitioning

DNN models are known to learn spurious correlations, which can affect their ability to generalize to data outside the training dataset^21,22^. Specifically, DNN models can learn inaccurate correlations by mistaking chance relationships between relevant and non-relevant features in a dataset as genuine indicators of object presence in an image^23,24^. In health-related applications, annotation artifacts that could serve as sources for spurious correlations in skin cancer classification were studied^25^. Similarly, findings on the effect of spurious correlations in pneumonia^26^ and COVID-19^27^ detection using chest radiographs have also been published.

To encourage the development of trustworthy DNN models that rely less on spurious correlations, we created a dataset partition of “negative images”, comprising microscopy images of non-infected blood samples. We obtained this partition of 93 images by applying blur and frame-differencing checks to all the extracted frames from the videos of non-infected blood samples, as illustrated in Fig. 1. The resulting partition provides an additional useful benchmark for evaluating the predictive performance of DNN models for trypanosomiasis detection. Ideally, a DNN model should not identify trypanosome parasites in the negative images; however, this may not always be true in practical settings. Consequently, if a DNN model predicts the presence of trypanosome parasites in the negative images, the associated probabilities are anticipated to be lower than those for microscopy images of infected blood samples. This would suggest that the DNN model possesses potential for practical application outside the data used to train it.

## Data Records

**Fig. 8:**
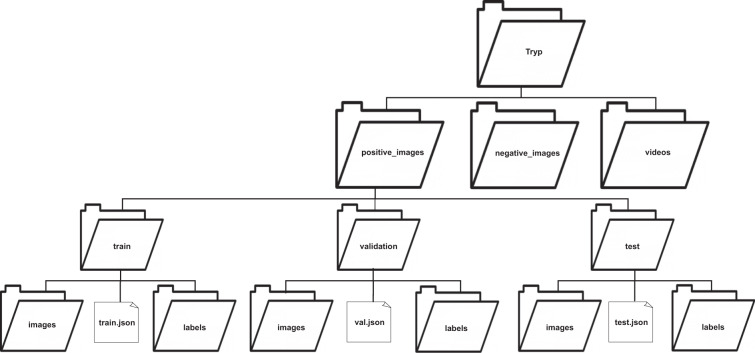
Directory structure for the Tryp dataset.

The Tryp dataset is available for download from figshare^28^. Notably, this dataset is distributed under a Creative Commons license, which fosters open access to scholarly resources. Decompression of *Tryp.zip* reveals three primary directories: *positive_images*, *negative_images*, and *videos*. The *videos* directory contains all the original captured videos from which the images in the Tryp dataset were extracted; these videos are grouped into positive and negative directories. Within *positive_images*, there are three sub-directories: *train*, *validation*, and *test*. Each *train*, *validation*, and *test* directory in-turn contains two sub-directories, *images* and *labels*, and a JSON file. The directories *images* and *labels* contain the images and annotation files, respectively, in the format compatible with the You Only Look Once version 7 (YOLOv7)^29,30^ model. The JSON files contain the corresponding annotations in the MS COCO format, suitable for training the Faster Region-based Convolutional Neural Networks (Faster R-CNN)^31^ and RetinaNet models. The naming format for the video and image files are <video type>_<video number>.extension and <video type>_<video number>_<frame number>.extension, respectively. The video type could be *positive_video* or *negative_video*. Examples of a negative video and image file are *negative_video_001.avi* and *negative_video_002_00000001.jpg*, while those for a positive video and image file are *positive_video_005.mp4* and *positive_video_005_00000073.jpg*. Figure 8 visualizes the structure of *Tryp.zip*. Additionally, Table 2 summarizes key characteristics of the Tryp dataset.

**Table 2:** A summary of the Tryp dataset characteristics, encompassing image and parasite counts across various partitions, as well as the distribution of images with respect to microscope type and image resolution.

Partition	Image Count	Parasite Count	IX83 Microscope	CKX53 Microscope	
			Resolution A	Resolution B	Resolution C
Train	1,893	27,489	392	1,068	433
Validation	610	8,697	120	347	143
Test	612	9,094	125	347	140
Negative Images	93	0	93	0	0

The resolutions denoted as Resolution A, Resolution B, and Resolution C correspond to 1,360 × 1,024, 1,920 × 1,080, and 720 × 404 pixels, respectively.

**Table 3:** Description of the 5-fold validation data.

		Fold				
		1	2	3	4	5
Number of Frames	Train	2671	2508	2087	2623	2539
	Validation	436	599	1020	484	568
Number of Videos	Train	64	65	65	65	65
	Validation	17	16	16	16	16

## Technical Validation

The development of the *Tryp* dataset involved methodology decisions to enhance its research utility. This section outlines the measures adopted to ensure quality, making the dataset a reliable asset for further research. We discuss the criteria for selecting object detection models, the model training, and the model evaluation processes tailored to align with the evolving needs of trypanosomiasis research. Finally, we provide our evaluation results.

### Model training and evaluation

#### Object detection models

In crafting a validation process for the Tryp dataset, the foremost consideration was the rich diversity encapsulated within this dataset. In alignment with this diversity, Faster R-CNN, RetinaNet, and YOLOv7 models were selected as a cross-section of prevalent methods in object detection literature. Our goal for selecting well-known models was to leverage their proven capabilities and to rigorously test whether they can achieve meaningful learning on the Tryp dataset.

#### Training process

The training process is inspired by the well-recognized practice of transfer learning^32^, particularly the fine-tuning technique^33^. For Faster R-CNN and RetinaNet, we fine-tuned models pre-trained on the MS COCO dataset^34^ from the Torchvision GitHub repository (https://github.com/pytorch/vision). The backbone network is a pre-trained ResNet50, and we did not freeze any layers in this backbone network during fine-tuning on Tryp. We used the CyclicLR learning rate (LR) scheduler of Torchvision with a base LR of 10^−5^, maximum LR of 5 × 10^−3^, *step_size_up* of twenty, and triangular2 mode to optimize the training process. The input to the Faster R-CNN and RetinaNet models is an image resized to maintain the original aspect ratio, with its smallest side at least 800 pixels and its largest side at most 1333 pixels. The input image to the YOLOv7 model is resized to 640 × 640 resolution. After each epoch during fine-tuning, we evaluated the prediction performance of the models on the validation dataset to select the best models. We fine-tuned for one hundred epochs with a batch size of eight, using two NVIDIA Titan RTX GPUs. The best models on the validation dataset partition were selected as the final models. For YOLOv7, we used the default configuration of the base model from the original implementation^30^ without any changes. The fine-tuning epochs and final model selection process mirror those used for Faster R-CNN and RetinaNet. The Faster R-CNN model took about eight hours to train, the RetinaNet model took about eight hours and thirty minutes, and the YOLOv7 model took about one hour and thirty minutes.

#### Evaluation process

We evaluated all three models utilizing the SVF partitioning strategy. However, due to limitations in computational resources and time, only the Faster R-CNN model was assessed using the SEV partitioning strategy and 5-fold cross-validation. The evaluation metrics included recall, precision, average precision at an intersection over the union of 50% (AP@IoU_0.5_), and the F1 score. Adopting these metrics was strategically aimed at facilitating a comprehensive and rigorous analysis. Collectively, these metrics offer a depth of insight that a single measure, such as AP, could not provide, thereby enabling a more nuanced and holistic evaluation of model performance.

## Results

Our goal of fine-tuning the selected object detection models on the Tryp dataset is to establish baseline performance and assess the viability of directly detecting the trypanosome parasite from unstained thick blood smear microscopy images. We provide these results under three evaluation settings: (1) performance on the validation and test dataset partitions of Tryp (refer to Fig. 1), (2) performance under 5-fold cross-validation evaluation using the SEV strategy, and (3) performance on the negative images–the negative images in Fig. 1. In our evaluation, we implemented a confidence threshold of 0.5, discarding predictions falling below this criterion. That way, we can mitigate the influence of improbable predictions, ensuring that our analysis prioritizes higher-confidence predictions. Furthermore, we can optimize the trade-off between precision and recall, yielding a more robust model performance evaluation.

### Performance on the validation and test partitions

**Fig. 9:**
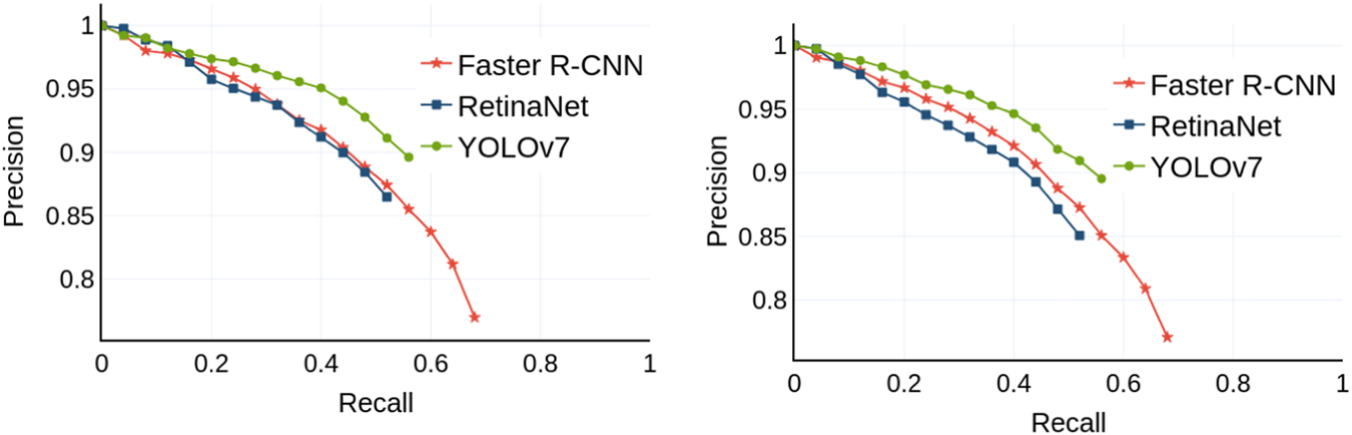
Precision-Recall curves for the detection performance of the evaluated models on the Tryp validation dataset partition (left) and the Tryp test dataset partition (right).

First, we present the AP, precision, recall, and F1 score results in Table 4. We show the results for both the validation dataset partition, which was used to select the best model during training, and the test dataset partition. The results are comparable, indicating no over-fitting under these settings. As shown in Table 4, the models have different performances, with YOLOv7 having the best precision and F1 score of 0.87 and 0.72, respectively. Faster R-CNN has the best AP and recall performance, obtaining a value of 0.71 for both metrics. The performance of the models can be further seen in the precision-recall curves in Fig. 9. Based on this presented figure, the YOLOv7 model achieves the highest precision among the compared models, while the Faster R-CNN model surpasses its counterparts in terms of recall.

**Table 4:** Performance of the evaluated models on the Tryp dataset.

Model	Dataset Partition	Performance Metrics at IoU = 0.5			
		AP	Precision	Recall	F1 score
Faster R-CNN	Validation	0.65	0.71	0.71	0.71
	Test	0.63	0.71	0.70	0.71
RetinaNet	Validation	0.52	0.83	0.56	0.67
	Test	0.50	0.82	0.55	0.66
YOLOv7	Validation	0.57	0.87	0.62	0.72
	Test	0.55	0.87	0.62	0.72

**Fig. 10:**
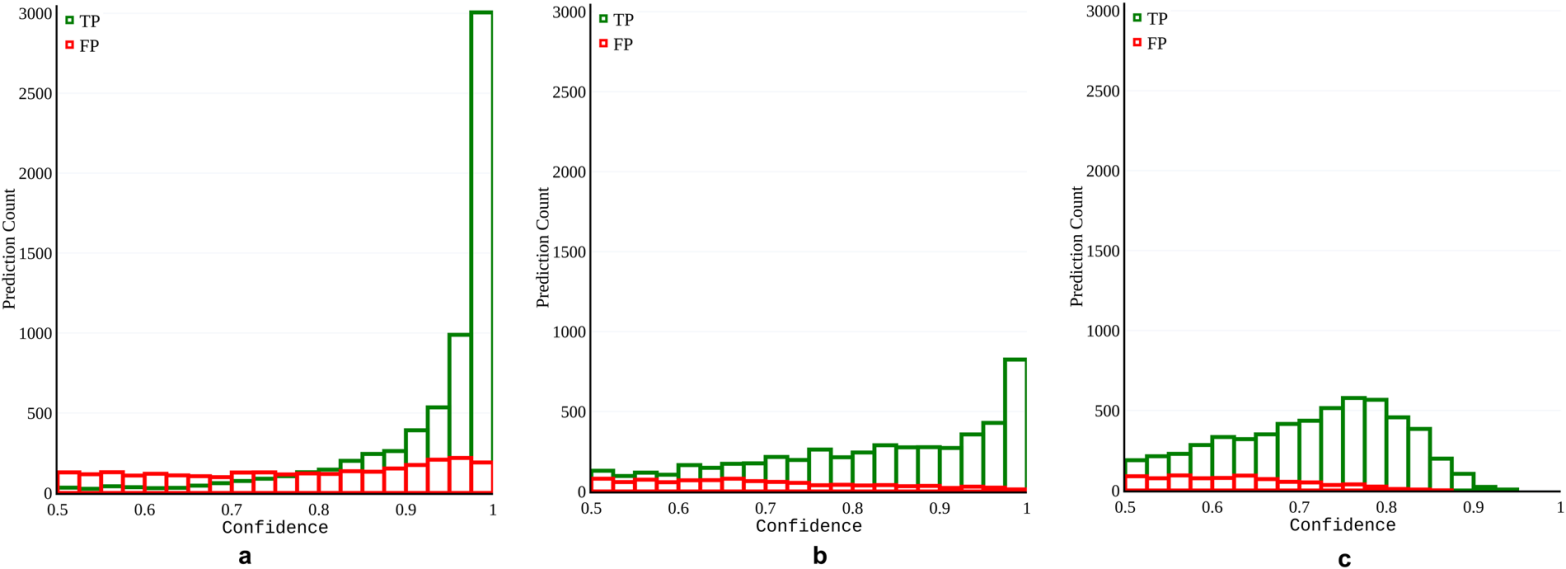
Confidence histograms of the true positive (TP) and false positive (FP) predictions for the test dataset partition of Tryp. The three histograms represent the confidence generated by (**a**) Faster R-CNN, (**b**) RetinaNet, and (**c**) YOLOv7.

Although informative, Table 4 does not provide information about the nature of the confidence the models assign to their predictions. We present this information as confidence histograms for all three models in Fig. 10. These histograms denote the counts of true positive and false positive predictions for each histogram bin. The figures show that all the models are likely to assign lower confidence to false positive predictions. While YOLOv7 is more conservative in assigning confidence values closer to one, it is also less likely to assign high confidence to false predictions. Similarly, RetinaNet is less likely to assign high confidence to false positive predictions; however, it is not as conservative in assigning high confidence to true positive predictions. Faster R-CNN has the highest prediction count, i.e., fewer false negative predictions than the other two model models. It is also more likely to assign higher confidence to false predictions.

### Performance under 5-fold cross-validation evaluation

Table 5 summarizes the 5-fold cross-validation performance of a Faster R-CNN model trained on the Tryp dataset. The model exhibited a variation in AP across the five folds, with values ranging from 0.44 to 0.72, resulting in a mean AP of 0.61 (std: 0.12). The precision of the model was relatively consistent, with values ranging from 0.69 to 0.86 and a mean precision of 0.79 (std: 0.06). The recall varied from 0.48 to 0.76, with a mean recall of 0.66 (std: 0.12). The F1 score, a measure of the model’s accuracy, ranged from 0.61 to 0.79, with a mean F1 score of 0.71 (std: 0.07). These results indicate that the model demonstrated reasonable and consistent performance across different folds in the 5-fold cross-validation evaluation, although there was some variation in recall and AP. We recommend the users of the Tryp dataset to additionally employ cross-validation, which presents a more realistic evaluation of model performance. The JSON files for the train and validation partitions of all the folds and the code to reproduce the 5-fold evaluation are provided in the code repository.

**Table 5:** 5-Fold cross-validation performance for the Faster R-CNN model on the Tryp dataset.

	AP	Precision	Recall	F1 score
Fold 0	0.72	0.82	0.76	0.79
Fold 1	0.63	0.69	0.70	0.70
Fold 2	0.70	0.78	0.76	0.77
Fold 3	0.55	0.78	0.62	0.69
Fold 4	0.44	0.86	0.48	0.61
Mean ± Std	0.61 ± 0.12	0.79 ± 0.06	0.66 ± 0.12	0.71 ± 0.07

### Performance on the negative images

**Fig. 11:**
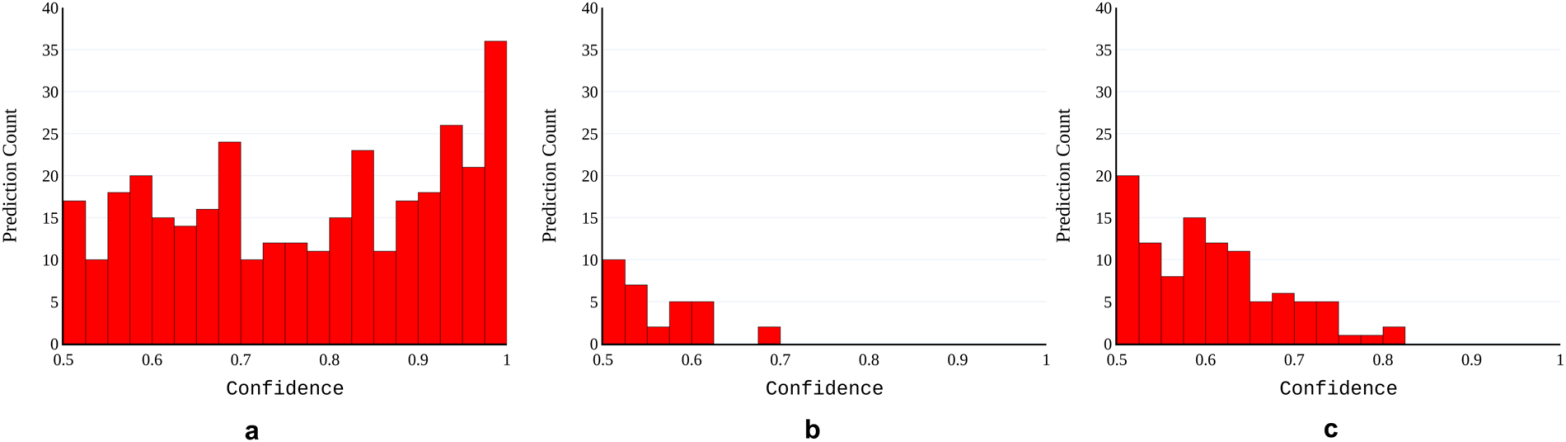
Confidence histograms for the predictions on the *negative images* partition of Tryp. The three histograms represent the confidence generated by (**a**) Faster R-CNN, (**b**) RetinaNet, and (**c**) YOLOv7.

The negative images (Fig. 1) are images from blood samples of non-infected mice. By evaluating our models on this dataset, we can observe (i) how likely the models are to predict the presence of parasites when there are no parasites and (ii) how likely they are to assign high confidence to false predictions. Faster R-CNN, RetinaNet, and YOLOv7 predicted 346, 31, and 103 parasite bounding boxes, respectively. The distribution of the assigned confidence is presented in Fig. 11. These plots indicate that RetinaNet is the least likely to predict bounding boxes for the negative images. Even when it does so, it is more likely to assign lower confidence than the two other models. YOLOv7 is the next better-performing model, while Faster R-CNN is the most likely to assign high confidence to false predictions. From this simple evaluation, we can infer that the Faster R-CNN model may have learned more features that may not necessarily indicate the presence of the parasites. More detailed experiments would be required to understand these preliminary results fully.

## Usage Notes

During the construction of the Tryp dataset, significant manpower and methodical efforts were dedicated to identifying and annotating the parasite. Through this process, we recognized that certain annotation decisions were inherently subjective, and it is plausible that a minor portion of parasites may have been overlooked. The enhancement of the quality of a dataset is an ongoing endeavor that necessitates continuous scrutiny and refinement. Future research in object detection could provide valuable insights by offering actionable feedback to dataset creators, such as pinpointing potential false positive bounding boxes that correspond to missed parasite annotations. Such feedback mechanisms could be instrumental in enhancing the quality of subsequent datasets.

Moreover, while many species of trypanosome parasites may present a morphology analogous to the one in our dataset under comparable microscopy capture processes, it would be imprudent to generalize the findings of this study to other species without further assessment. Thus, there remains a significant opportunity for future investigations focusing on the out-of-domain generalization that might arise from the application of the Tryp dataset and other datasets that may be developed in the future.

## Data Availability

The code and detailed documentation on how to use it to reproduce the results presented in this study is publicly available at https://github.com/esla/trypanosome_parasite_detection under the permissive Berkeley Software Distribution (BSD) 3-Clause license.
